# Editorial: Intraoperative Neuromonitoring: Evaluating the Role of Continuous IONM and Intermittent IONM in Emerging Surgical and Percutaneous Procedures

**DOI:** 10.3389/fendo.2022.960633

**Published:** 2022-07-12

**Authors:** Stefano Spiezia, Chiara Offi, Claudia Misso, Maria Gaudiello

**Affiliations:** Department of Endocrine Surgery, Local Health Authority Naples 1 Center, Naples, Italy

**Keywords:** thyroid neuromonitoring, IONM (intraoperative neuromonitoring), thyroid surgery, thermoablative procedures, robotic surgery

## Introduction

The first anatomical description of the recurrent laryngeal nerve (RLN) is due to Galen in the 2nd century AD, but only in the last century its importance has been understood by endocrine surgeons. RLN injury is a major complication of thyroid surgery and leads to vocal cord paralysis (VCP). RLN’s visualization is the gold standard for integrity preservation. However, an anatomical intact nerve does not represent its functionality. Reviews of large case series have shown a unilateral VCP in 9.5%, while bilateral VCP in 1.3%. Instead, the permanent VCP rate is 1% –2%. Unilateral VCP can cause hoarseness, while bilateral VCP is associated with severe respiratory distress and requires a tracheostomy. However, both are associated with a significant impact on the patient’s quality of life, on the cost for the National Sanitary System and may have medico-legal consequences.

Intraoperative neuromonitoring (IONM) has been developed on the principle of RLN and superior laryngeal nerve (SLN) identification. IONM helps the surgeon in nerves identification in order to reduce paralysis rates. In addition, it is used in the identification of clamping, ligation, compression, traction, thermal injury, or ischemia injuries, in which the RLN appears anatomically intact, but functionally damaged.

Intermittent (I-IONM) and continuous (C-IONM) intraoperative neuromonitoring can be considered an additional technique to the routine practice of visual identification of the RLN and SLN during thyroid surgery.

Many surgical societies and guidelines recommend the use of IONM, especially in surgery for recurrent cancer, locally advanced cancer or large goiters. However, Literature shows discordant results.

In C-IONM, nerve stimulation occurs throughout the endocrine procedure which provides a constant evaluation of nerve and vocal cord functional integrity. The probe can be applied directly to the vagus nerve. C-IONM methodologies allow potentially harmful surgical maneuvers to be modified in real time and thus has a role in the prevention and prediction of nerve damage.

We propose an editorial on Frontiers focusing on the experience of different authors with the use of C-IONM and I-IONM. This edition would, also, addresses the use of neuromonitoring in novel surgical approaches (Robotic surgery, minimally invasive technique and thermoablation techniques). Pace-Asciak et al. (2022) publish a review on C-IONM and I-IONM and on the use of intraoperative nerve monitoring technologies in scarless thyroid surgery and percutaneous approaches to thyroid pathology. It emerges that the role of I-IONM and C-IONM in thyroid surgery is an adjunct to determining the integrity of the RLN and the external branch of SLN. Mazzone et al. (2021) evaluate electromyographic waveforms related to vagus monitoring. The authors, according to Literature, affirm that an amplitude ≥500 µV can be considered as a RLN damage. The next frontier is the evolution of these technologies within the confines of endoscopic and robotic surgery as well as radiofrequency ablation. In robotic surgery, Zhang et al. (2022) suggest that the percutaneous C-IONM in robotic bilateral axillary thyroid surgery is feasible in all cases. The study declares that the C-IONM in robotic thyroid surgery allows uninterrupted real-time monitoring of RLN function through continuous stimulation of the vagus nerve. In minimally invasive approaches, Kuo et al. (2021) suggest that IONM has not been routinely applied in early experience with the transoral endoscopic thyroidectomy vestibular approach (TOETVA). The authors calculated a learning curve of 35 cases to reduce the complication’s rate. Furthermore, McManus and Kuo (2022) publish a review on the use of IONM in RFA including the cutaneous surface electrode system. The review concludes that the RFA’s IONM is possible in local anesthesia with cutaneous surface electrodes. The protection of the recurrent laryngeal and vagus nerves during RFA requires an understanding of neck anatomy and the ability to infer the course of the RLN and vagus nerves using sonography. Finally, Yu et al. (2022) propose a technique for preserving the RLN course. They suggest the intraoperative neural tunnel protecting (INTP) to reduce the incidence of RLN damage in open, trans breast, and transoral endoscopic thyroidectomy.

Today, IONM is part of the common clinical practice of endocrine surgeons. In the United States, it is used by 53% of general surgeons and 65% of otolaryngologists. Numerous international guidelines suggest the routine use of the IOMN. The German Guidelines and the International Neural Monitoring Study Group Recommendation (INMSG) suggest the use of IONM in all thyroid and parathyroid surgeries. The Guidelines of the American Academy of Otolaryngology - Head and Neck Surgery (AAO - HNS) suggest the use of IOMM in cases of bilateral thyroid surgery, totalization and in existing cases of VCP. The American Thyroid Association (ATA) guidelines suggest the use of the IOMM as, in cases of outpatient surgery, it can give indications on neuronal functioning and facilitates discharge planning. Also, the guidelines of the American Head and Neck Society (AHNS) suggest that IOMM is important in cases of thyroid cancer.

Nerve lesions during thyroid surgery can be diagnosed directly with IONM and indirectly with postoperative fibrolaryngoscopy. Obviously, the lesion’s severity varies according to the number of damaged fibers or the involvement of the myelin sheath. The function’s recovery requires a time ranging from minutes to months. IONM can therefore be considered a safe technique since when the wave shape is preserved, in cases of LOS, the functionality of the vocal cord in the postoperative period is preserved. This is of fundamental importance in the two-stage planning of total thyroidectomy.

According to the ATA Guidelines, all patients undergoing thyroid and parathyroid surgery must undergo preoperative fibrolaryngoscopic examination. The preoperative evaluation helps to identify pre-existing paralysis or paresis, facilitating the surgeon in the diagnosis and in the planning of surgery.

In conclusion, IONM is a valuable technique, which adds information to the RLN and SLN integrity. IONM helps in defining nerve anatomy, predicting prognosis and intraoperative management. Further studies are needed on I- and C-IONM.

## Author Contributions

All authors contributed significantly to the present research and reviewed the entire manuscript. SS: Participated substantially in conception, design and execution of the study and in the analysis and interpretation of the data; also participated substantially in the drafting and editing of the manuscript. CO: Participated substantially in conception, design and execution of the study and in the analysis and interpretation of the data; also participated substantially in the drafting and editing of the manuscript. CM: Participated substantially in conception and design of the manuscript. MG: Participated substantially in conception and design of the manuscript. All authors have read an approved the final manuscript.

## Conflict of Interest

The authors declare that the research was conducted in the absence of any commercial or financial relationships that could be construed as a potential conflict of interest.

## Publisher’s Note

All claims expressed in this article are solely those of the authors and do not necessarily represent those of their affiliated organizations, or those of the publisher, the editors and the reviewers. Any product that may be evaluated in this article, or claim that may be made by its manufacturer, is not guaranteed or endorsed by the publisher.

